# Unexpected frequency of genomic alterations in histologically normal colonic tissue from colon cancer patients

**DOI:** 10.1007/s13277-016-5181-0

**Published:** 2016-08-02

**Authors:** Donatella Conconi, Serena Redaelli, Giorgio Bovo, Biagio Eugenio Leone, Emanuela Filippi, Luciana Ambrosiani, Maria Grazia Cerrito, Emanuela Grassilli, Roberto Giovannoni, Leda Dalprà, Marialuisa Lavitrano

**Affiliations:** 1 School of Medicine and Surgery, University of Milano-Bicocca, via Cadore 48, 20900 Monza, Italy; 2 Unit of Pathology, San Gerardo Hospital, Monza, Italy; 3 Section of Pathology, Desio Hospital, Desio, Italy; 4 Department of Pathology, Valduce Hospital, Como, Italy; 5 Medical Genetics Laboratory, San Gerardo Hospital, Monza, Italy

**Keywords:** Colorectal cancer, Copy number alterations, Histologically normal tissue, Precancerous conditions

## Abstract

As shown by genomic studies, colorectal cancer (CRC) is a highly heterogeneous disease, where copy number alterations (CNAs) may greatly vary among different patients. To explore whether CNAs may be present also in histologically normal tissues from patients affected by CRC, we performed CGH + SNP Microarray on 15 paired tumoral and normal samples. Here, we report for the first time the occurrence of CNAs as a common feature of the histologically normal tissue from CRC patients, particularly CNAs affecting different oncogenes and tumor-suppressor genes, including some not previously reported in CRC and others known as being involved in tumor progression. Moreover, from the comparison of normal vs paired tumoral tissue, we were able to identify three groups: samples with an increased number of CNAs in tumoral vs normal tissue, samples with a similar number of CNAs in both tissues, and samples with a decrease of CNAs in tumoral vs normal tissue, which may be likely due to a selection of the cell population within the tumor. In conclusion, our approach allowed us to uncover for the first time an unexpected frequency of genetic alteration in normal tissue, suggesting that tumorigenic genetic lesions are already present in histologically normal colonic tissue and that the use in array comparative genomic hybridization (CGH) studies of normal samples as reference for the paired tumors can lead to misrepresented genomic data, which may be incomplete or limited, especially if used for the research of target molecules for personalized therapy and for the possible correlation with clinical outcome.

**Electronic supplementary material:**

The online version of this article (doi:10.1007/s13277-016-5181-0) contains supplementary material, which is available to authorized users.

## Introduction

Colorectal cancer (CRC) is a heterogeneous disease defined by distinct molecular signatures, distinct pathological features, and distinct natural histories. There are at least three major molecular pathways to CRC genetically identified by chromosomal instability (CIN), microsatellite instability (MSI), and the CpG island methylator phenotype (CIMP). The CIN pathway is characterized by structural rearrangements, amplifications, deletions, and copy number alterations (CNAs). The MSI pathway’s main feature is the mutation or aberrant methylation of DNA mismatch repair genes, whereas in the CIMP pathway, excessive promoter methylation occurs [1].

Several groups have studied chromosomal aberrations in CRC by means of array comparative genomic hybridization (array-CGH) being the most common reported aberration gains on chromosomes 20q, 13q, 7p, and 8q and losses on chromosomes 18q, 17p, and 8p [2–4].

More recently, the increase in genomic studies lead to the identification of copy number aberrations linked to adenoma-carcinoma progression or linked to metastases. For example, gains on chromosomes 7p, 13q, and 20p/q and loss on 18q were reported as early DNA alterations [5]. Gains at 8q, 13q, and 20p/q and losses at 8p, 15p, 17p, and 18q were shown to be significantly changed between adenomas and stage I carcinomas [6].

Copy numbers of 3q, 10q, 11q, and 15q and Xp regions were reported as being linked to node metastases [7], whereas gains at chromosomes 1q, 6p21, 10p, and 17q21 and loss at chromosome 8p12 occurred more frequently in metastases than in primary tumors [6]. In addition, it has been investigated if shared CNAs may contain candidate genes for targeted therapy [8] or may have a possible correlation with clinical outcome. For example, losses at 16p13.3 and 19q13.3 were correlated with negative outcome, with a strong association with early relapse and death [6]. Also, loss of 8p23-p21 occurred more often in patients with a poor prognosis [9].

While several array-CGH analyses have been performed on colonic tumoral tissues, genetic analysis of histologically normal tissues is still lacking. In fact, in all the studies reported so far, colon normal tissues were used individually or pooled as reference for tumor but were never analyzed in itself, comparing different individual samples. Only one report shows small gains at 19q and 20q in pooled normal tissues [9]. Notably, in 10–20 % of CRC patients undergoing surgery with curative intent, a local recurrence successively occurs [10], suggesting that tumorigenic genetic lesions may be already present also when the colonic tissue appears histologically normal. If this was the case, the use in array-CGH studies of normal samples as reference for the corresponding tumors would lead to misrepresented genomic data, due to the loss of identical CNAs between the two samples. An additional consequence would also be that even available array-CGH data may be incomplete or limited, both for the research of target molecules for personalized therapy and for the possible correlation with clinical outcome.

To address this very important issue, we analyzed by CGH + SNP Microarray the genome of 15 paired tumoral and histologically normal colonic tissues. Here, we report for the first time the occurrence of CNAs as a common feature of the normal tissue from CRC cancer patients and discuss its significance.

## Materials and methods

### Samples

A total of 15 paired samples of sporadic tumoral and surrounding histologically normal tissue (taken at a distance of 10 cm from the tumor) were obtained from patients undergoing surgery in three different centers. University of Milano-Bicocca Ethics Committee approval and written informed consent was obtained before tissue collection, but blood samples were not included. Staging and grading were done according to the World Health Organization Consensus Classification.

### Array-CGH

For the array-CGH analysis, genomic DNA was extracted from fresh biopsies after enzymatic digestion with trypsin/EDTA 1X (EuroClone) at 37 °C for 60–90 min and proteinase K (Roche) at 37 °C for 30 min and purified using phenol/chloroform (Carlo Erba). Sample preparation, slide hybridization, and analysis were performed using CytoSure ISCA + SNP array 4x180K and CytoSure Cancer + SNP array 4x180K (Oxford Gene Technology, CGH and SNP probes provided on the same slide) according to the manufacturer’s instructions. Sex-matched commercial DNA samples (Promega) were used as reference DNA for normal samples, while normal paratumoral DNAs were used as reference for tumoral tissues. Tumoral samples were checked for the presence of at least 70 % of tumor epithelial cells by an expert pathologist on a slide of neighboring tissue. We collected fresh, consecutive, and unselected samples to perform array-CGH immediately after the histological diagnosis, without other information.

DNA extraction from normal and tumoral paired fresh samples received from the different hospitals was carried out in our laboratory, where all the DNA quality control checks required by array-CGH protocol were performed at every step: after the purification with phenol/chloroform, after enzymatic digestion, and after labeling. No differences were observed among samples from different hospitals. All samples showed a suitable quality for the protocol standards.

Briefly, the test and reference DNAs were denatured and labeled by random priming with Cy3-dUTP and Cy5-dUTP, respectively. Then, both samples were concentrated and then coupled according to the efficiency of labeling (only for commercial and normal DNA). Following probe denaturation and preannealing with *Cot-1* DNA, hybridization was performed at 65 °C with rotation for 40 h at 20 rpm.

The arrays were scanned at 2-μm resolution using an Agilent microarray scanner and analyzed using CytoSure Interpret Software.

Experiments showed excellent DLRS values (mean 0.18, range 0.15–0.31), so mosaic CNAs were also included in the study with a good confidence. To calculate the estimated percentage of mosaicism, we used the formula determined by Cheung et al. [11].

We applied a filtering option of a minimum of four aberrant consecutive probes and a minimum absolute average log2 ratio that differs among all samples and depends to DLRS values, so it is related to the quality of experiment. In particular, non mosaic gains and losses are identified by standard log2 ratio values for all samples: values over 0.6, which correspond to three copies, identify non-mosaic gains; values under −1, which correspond to 1 copy, identify non-mosaic losses. Accordingly, log2 ratio values for mosaic gains range between the DLRS value and 0.6 and for mosaic losses between the DLRS value and −1. The loss of heterozygosity (LOH) regions were selected with a score above 200 by CytoSure Interpret Software.

### Gene ontology analysis

To identify the most represented ontology classes among the genes contained in normal gained regions, we used the GOstat software [12]. The GO terms in the output are linked to a visualization tool for the GO hierarchy (AmiGO, the Gene Ontology database, version 1.8).

## Results

### General data

We performed array-CGH analysis on 15 paired tumoral and histologically normal samples obtained from patients affected by CRC. Males and females were equally represented: eight females and seven males. The mean age was 71.9 (range 52–85 years) and the majority of samples were adenocarcinomas (12 of 15). Tumors were mostly located in the ascending colon and hepatic flexure (40 %), following by sigmoid colon, descending colon, and rectum. The samples were almost equally distributed between stage II and stage III and between grade 2 and grade 3 (Table 1).

**Table 1:** Clinicopathologic characteristics of tumoral samples

Patient	Age	Sex	Histotype	Location	Stage	Grade
1	80	F	Adenocarcinoma	Sigmoid	IIA	2
2	71	F	Adenocarcinoma	Ascending colon and hepatic flexure	III	3
3	80	M	Undifferentiated carcinoma	Ascending colon and hepatic flexure	IIIA	3
4	59	F	Adenocarcinoma	Ascending colon and hepatic flexure	II	2
5	79	F	Adenocarcinoma	Ileocecal valve	IIIC	2
6	69	F	Adenocarcinoma	Ascending colon and hepatic flexure	II	3
7	85	M	Adenocarcinoma	Ascending colon and hepatic flexure	III	3
8	69	F	Adenocarcinoma	Sigmoid	IV	3
9	71	M	Adenocarcinoma	Cecum	IIA	2
10	77	M	Mucinous carcinoma	Rectum	IVA	nd
11	81	M	Adenocarcinoma	Sigmoid	IIA	2
12	85	F	Adenocarcinoma	Descending colon and splenic flexure	II	2
13	58	M	Adenocarcinoma	Rectum	IIIC	3
14	52	M	Adenocarcinoma	Descending colon and splenic flexure	III	2
15	62	F	Undifferentiated carcinoma	Ascending colon and hepatic flexure	IIA	4

Histotype, location, stage and grade are indicated

### Analysis of normal tissues

The CNAs of histologically normal samples greatly varied among different patients ranging from 26 to 345 (average 160). Comparing the number of CNAs detected in normal tissues vs reference DNA, a statistically significant difference was evident between stage II and stage III samples (*p* < 0.05, Student’s *t* test, staging is referred to the corresponding tumor sample), which was not present when samples were divided by grade or by tumor location (data not shown).

**Fig. 1:**
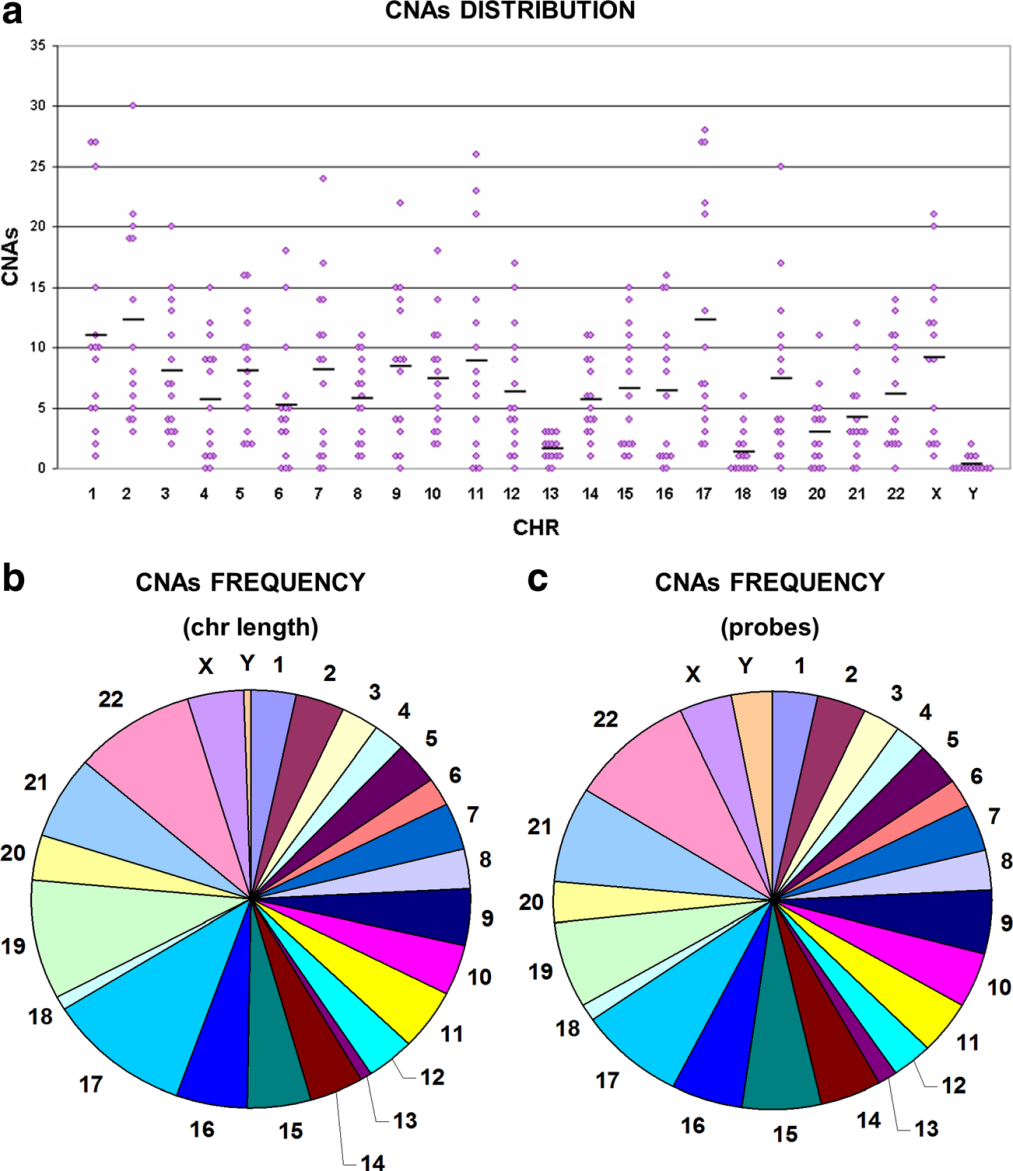
Copy number alterations in normal samples. Chromosome distribution of copy number alterations in normal samples (**a**) and CNA frequency over the chromosome length (**b**) and over the number of probes (**c**). Gains and losses are mixed together to assess which chromosomes were most affected by CNAs

CNA distribution within chromosomes was also variable, being the chromosomes with a higher average number of CNAs chr2, chr17, and chr1 (Fig. 1a). Calculating the density of CNAs over the chromosome length, chr17, chr19, chr21, and chr22 turned out as those with greater density, whereas chr13 and chr18 where those with fewer alterations (Fig. 1b). The same results were obtained calculating the density over the number of probes present on each chromosome (Fig. 1c).

Due to the high number of CNAs detected in the normal samples, we choose to further analyze those shared by at least 7 out of 15 cases. Following this approach, 90 CNAs were selected, mainly distributed on chromosomes 2 (8 CNAs), X (7 CNAs), 5, 7, 8, 10, and 17 (6 CNAs each). No shared CNAs were found on chromosomes 6, 18, and Y (Table S1). Notably, the majority of CNAs were mosaic copy number alterations.

**Fig. 2:**
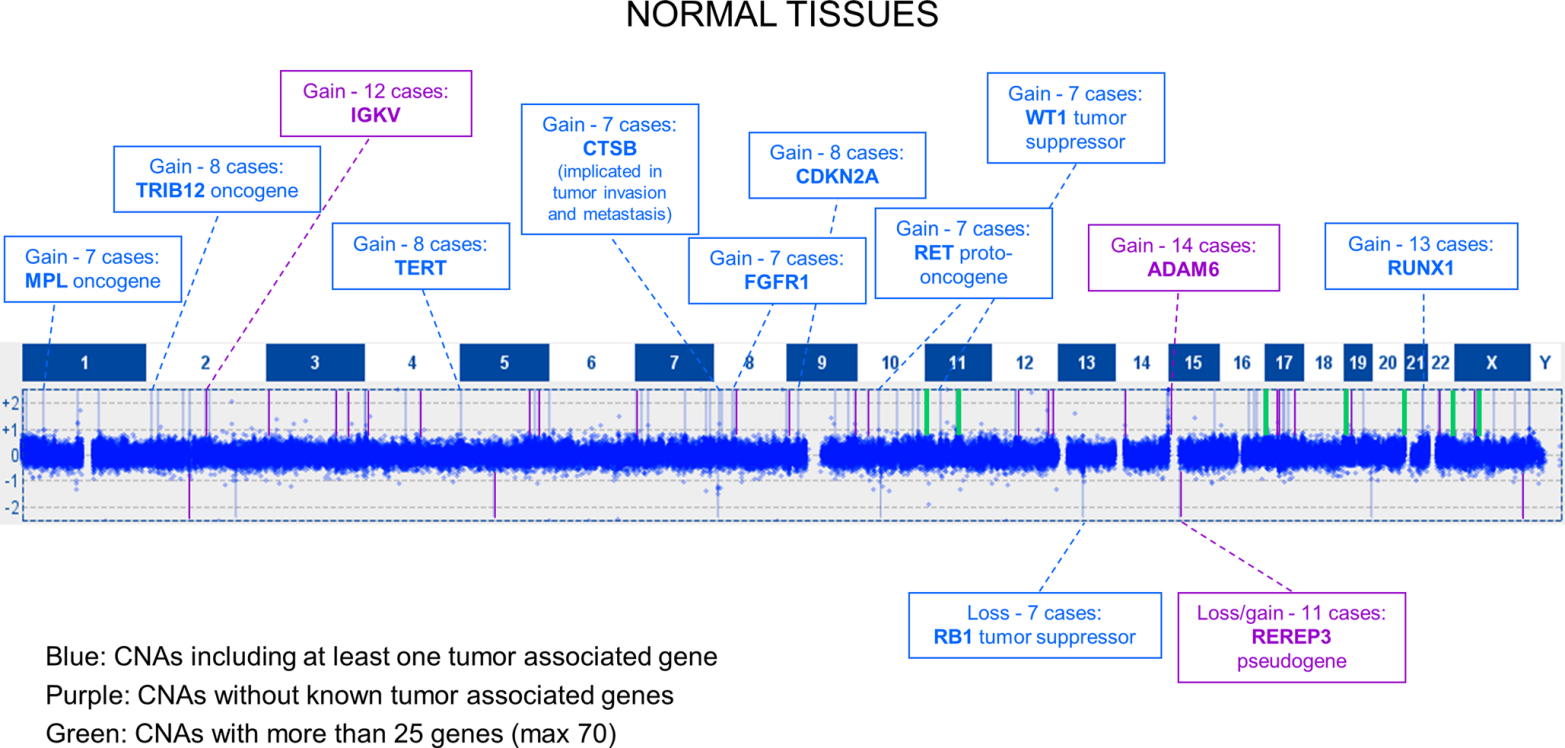
Distribution of shared CNAs in histologically normal tissues. Graphic representation of copy number alterations, detected by CGH, shared by at least 7 out of 15 normal tissues. Shared CNAs could be divided into three types: CNAs containing at least one tumor-associated gene (*blue*); CNAs not affecting known tumor-associated genes (*purple*); large-sized CNAs involving several genes (*green*)

Shared CNAs could be divided into three types: (1) CNAs containing at least one tumor-associated gene (Fig. 2), (2) CNAs not affecting known tumor-associated genes, (3) large-sized CNAs (large aberrations) involving several genes.

Interestingly, 13/15 samples showed a gain on 21q22.12 containing *RUNX1*, a gene involved in translocations in different types of leukemia and generally considered a tumor suppressor in myeloid neoplasms. Among the CNAs shared by the great majority of the samples, there are also two gains that were reported as polymorphic in the Database of Genomic Variants (DGV), hence devoid of a pathogenetic role: a non-mosaic gain, localized on 14q32.33, containing *KIAA0125* and *ADAM6* genes, shared by 14/15 samples and one containing *IGKV* present in 12 cases. A large group of gains, shared by approximately half of the samples, included the oncogenes *MPL* (1p34.2, 7 samples), *TRIB2* (2p24.3, 8 samples), *ERBB2* (17q12, 7 samples), and *ARAF* (Xp11.23, 7 samples) and other genes implicated in oncogenesis such as *TERT* (5p15.33, 8 samples), *FGFR1* (8p11.23-p11.22, 7 samples), and *RECQL4* (8q24.3, 9 samples). Tumor suppressor genes such as *PTCH1*, *DAB2IP*, and *WT1*, usually lost in tumors, were also included in the gained regions of approximately half of the samples. Also, *RET* (10q11.21, seven samples), *CTSB* (8p23.1, seven samples), and *CDKN2A* (9p21.3, seven samples) copy number gains were found, but they are included in DGV as benign variants.

To note, the only two normal samples, whose paired tumoral tissue has an undifferentiated histotype, shared two gains not present in any other normal sample, one on chromosome 8q24.21 including *MYC* oncogene and one on chromosome Xp21.3 containing the *MAGEB18*, *MAGEB6*, and *MAGEB5* genes.

The copy number gain of *ERBB2*, *RECQL4*, and *MYC* oncogene and *MAGEB* family has been frequently reported in colorectal carcinoma, as shown in a recent review [13]. Twelve shared CNAs fell into the second group that does not contain any known tumor-associated gene.

Next, in order to find statistically overrepresented GO categories among the genes included in the gained regions (Fig. S1), we performed a gene ontology analysis, using the GOstat software. Table S2 shows statistically significant (*p* < 0.05) GO terms. The most represented category was regulation of the biological process which comprises regulation of transcription, of gene expression, of growth, of apoptosis and of cellular metabolic process.

### Analysis of tumoral tissues

**Fig. 3:**
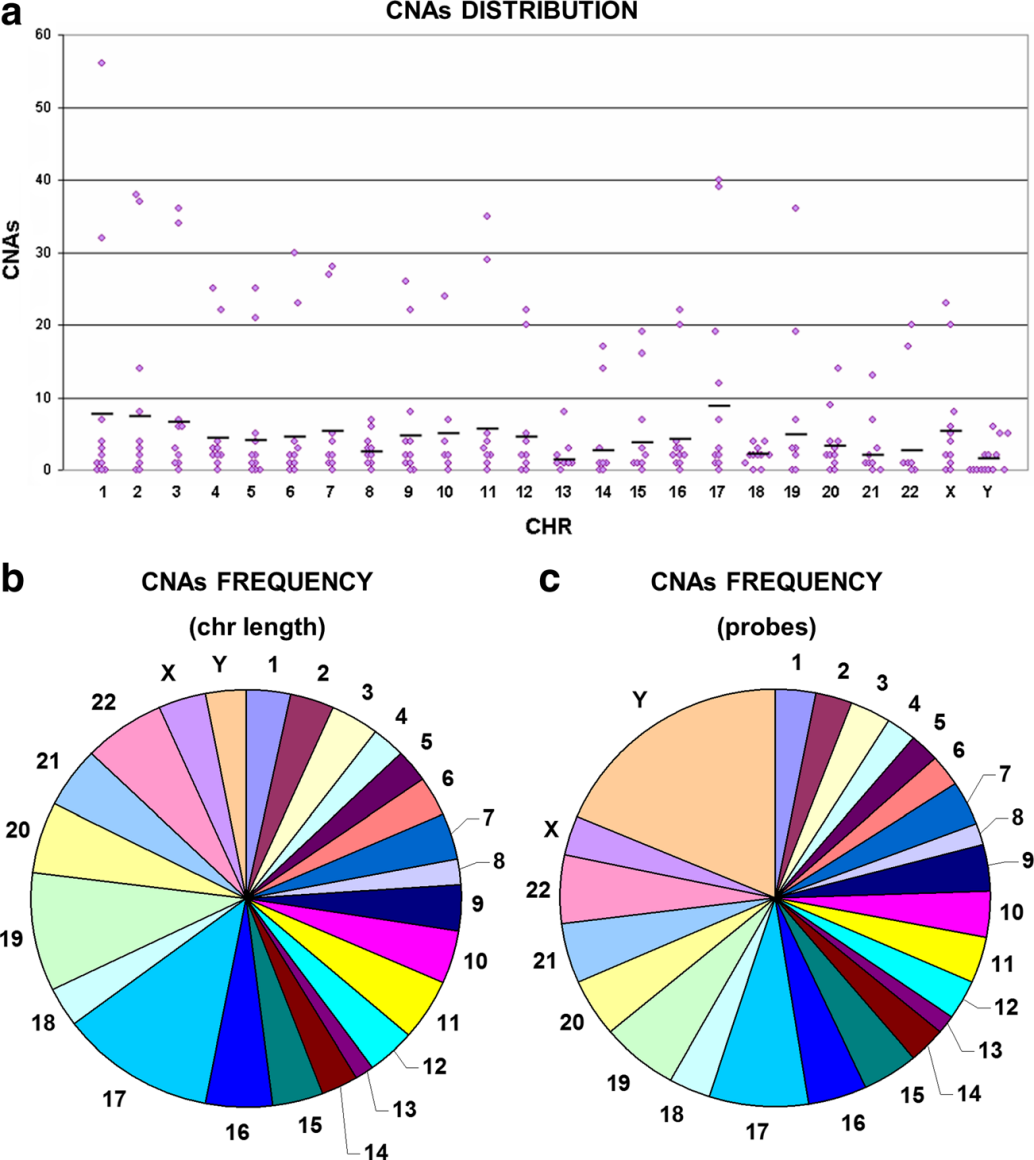
Copy number alterations in tumoral samples. Chromosome distribution of copy number alterations in tumoral samples (**a**) and CNA frequency over the chromosome length (**b**) and over the number of probes (**c**). Gains and losses are mixed together to assess which chromosomes were most affected by CNAs

Tumoral samples, tested against the paired normal sample, showed a lower average but a more widespread ranging of CNAs (5 to 549, average 106). Comparing the number of CNAs in different tumoral samples (excluding the two samples with a higher number of CNAs), a statistically significant difference was evident between stage II and stage III samples and between stage IV and stage III samples (*p* < 0.05, Student’s *t* test), but it was not present when the samples were divided by grade or by tumor location (data not shown). CNA distribution within the chromosomes was variable, being chr17, chr1, and chr2 those with a higher average number of CNAs (Fig. 3a). Chromosomes with the greatest density of CNAs over the chromosome length were chr17, chr19, and chr22, whereas chr8 and chr13 were those with the fewest alterations (Fig. 3b). The same results were obtained calculating the density over the number of probes present on each chromosome, except for Y showing a strong frequency increase, probably due to the small number of probes on this chromosome (Fig. 3c).

**Fig. 4:**
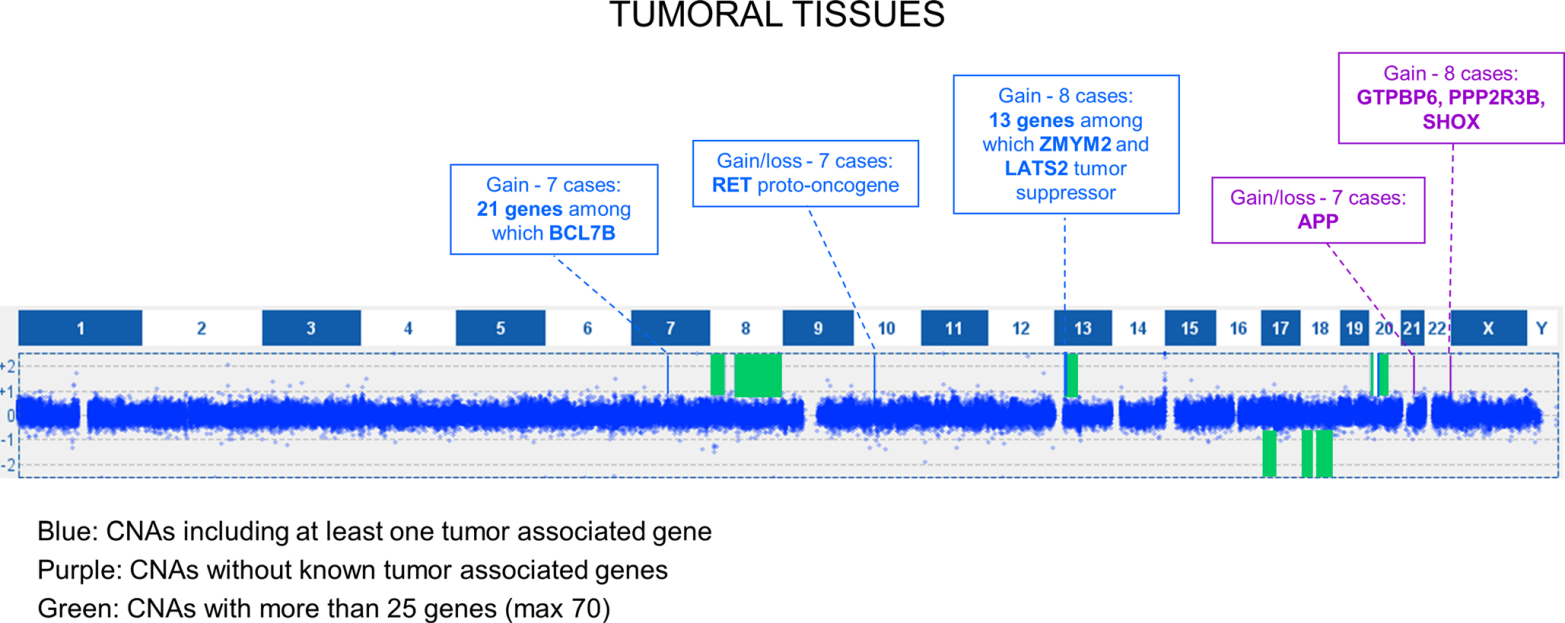
Distribution of shared CNAs in tumoral tissues. Graphic representation of copy number alterations, detected by CGH, shared by at least 7 out of 15 tumoral tissues. Shared CNAs could be divided into three types: CNAs containing at least one tumor-associated gene (*blue*); CNAs not affecting known tumor-associated genes (*purple*); large-sized CNAs involving several genes (*green*)

Also for tumoral samples, we choose to further analyze CNAs shared by at least 7 out of 15 cases: this led to the selection of 12 CNAs, mainly consisting in large aberrations encompassing several genes (Fig. 4); notably, the majority were mosaic CNAs. The most shared CNA (10/15) was a gain of 20q11.21-q11.22, which in eight samples was larger and extended from 20q11.21 to 20q13.33. The second most shared CNA was located in 20p13 and was found in 9/15 samples. To note, the only sample with mucinous histotype had an opposite CNA, with a loss of 20p and 20q regions. Among the most shared CNAs, two were characterized by being in loss in some samples and in gain in others: a CNA on chromosome 10 containing *RET* oncogene, shared by eight samples and a CNA with *APP*, shared by seven samples. Finally, in seven samples, we found on chromosome 7 a shared gained region containing several genes.

### Comparison between histologically normal and tumoral tissues

#### Copy number alterations

We divided CNAs into unique or shared between paired normal and tumoral tissue as listed in Table 2. The number of shared CNAs was not identical because some large CNAs can contain smaller CNAs in the same region. Using normal tissue as a reference for the corresponding tumor, alterations that are equal between both tissues are not detectable by array-CGH, since the competition for the oligonucleotide probes is the same and only different ones can be identified. As a consequence, CNAs present in both normal and tumoral samples (shared CNAs) must be more amplified in the latter; otherwise, they would have been scored as disomic.

**Table 2:** Copy number alterations of tumoral and normal samples

Patient	Normal aberrations			Tumoral aberrations		
	*Total*	*Unique*	*Shared*	*Total*	*Unique*	*Shared*
1	55	45	10	46	38	8
2	239	45	194	549	323	226
3	178	175	3	5	4	1
4	174	147	27	61	40	21
5	44	28	16	28	19	9
6	221	38	183	471	277	194
7	295	202	93	25	5	20
8	39	22	17	91	78	13
9	26	12	14	54	45	9
10	60	36	24	92	78	14
11	71	40	31	54	39	15
12	124	105	19	42	34	8
13	336	327	9	13	9	4
14	389	359	30	18	10	8
15	151	131	20	34	26	8

Excluding “masked” CNAs, our results highlighted three groups: (1) samples with an increased number of CNAs in tumoral vs normal tissue (five cases); (2) samples with a similar number of CNAs in both tissues (two cases); (3) samples with a decrease of CNAs in tumoral vs normal tissue (eight cases). Samples falling in the first group, such as those from patients 2 and 6, are characterized by increased genomic instability. Instead, in samples of the third group, a decreased genomic instability was evident (for example patients 3, 12, and 14), probably due to a selection of the cell population in tumoral tissues. From this last group, we analyzed CNAs present only in the tumoral tissue (unique) and disomic in the paired normal sample, assuming a possible role in tumor progression; only two CNAs shared by at least three of eight samples met this parameter: one comprising *RBFOX1* gene and the other comprising *PCDH11Y* gene. *RBFOX1* copy number gain and loss are reported in DGV as benign variants.

Finally, we analyzed for all samples the CNAs shared between normal and tumoral samples. Eight gains were shared by at least three samples, localized in the following: 2p24.3 (*TRIB2* gene), 3q29 (*MFI2*), 10q11.21 (*RET*), 12q13.12 (*WNT1*, *DDN*, *PRKAG1*), 21q22.12 (*RUNX1*), Xq21.1 (*BRWD3*), Xq25 (*GRIA3*), Xq25 (*XIAP*). Most of these genes were reported to be involved in several types of cancer (Table 3). Gene ontology analysis showed different significant ontology classes but includes no more than one gene.

**Table 3:** Gains shared by at least three samples

Patients	Gain/loss	Genes
1, 2, 6	Gain 2p24.3	*TRIB2* (oncogene that inactivates the transcription factor C/EBPalpha and causes acute myelogenous leukemia)
2, 4, 6	Gain 3q29	*MFI2* (cell-surface glycoprotein found on melanoma cells)
2, 4, 6	Gain 10q11.21	*RET* (Proto-oncogene. Diseases associated include familial papillary thyroid carcinoma)
2, 4, 6	Gain 12q13.12	*WNT1* (secreted signaling protein. Implicated in oncogenesis), *DDN* (promotes apoptosis of kidney glomerular podocytes), *PRKAG1* (Regulatory subunit of the AMP-activated protein kinase)
1, 2, 6	Gain 21q22.12	*RUNX1* (involved in the development of normal hematopoiesis. Diseases associated include familial platelet disorder with associated myeloid malignancy)
2, 4, 6	Gain Xq21.1	*BRWD3* (chromatin-modifying function. Diseases associated include chronic lymphocytic leukemia)
2, 4, 6	Gain Xq25	*GRIA3* (receptor for glutamate)
2, 4, 6	Gain Xq25	*XIAP* (oncogene. Diseases associated include chronic neutrophilic leukemia)

#### Aneuploidies

By using the Cytosure software (that gives the possibility to obtain a graphical representation of averages and standard deviations of all log2 ratios of chromosome probes), we evaluated the distribution of the chromosome probes and identified the aneuploidies, also at mosaic (Fig. S2).

**Fig. 5:**
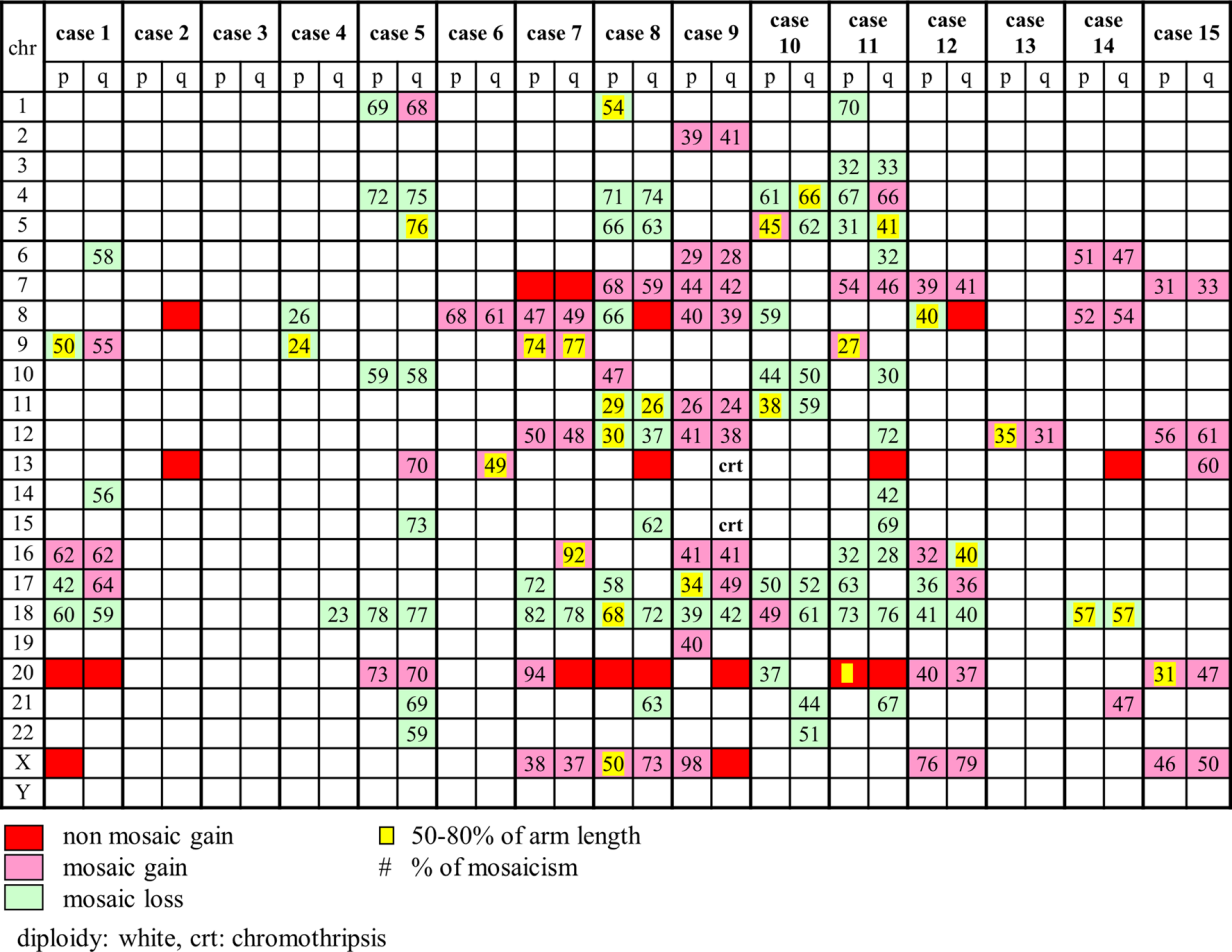
Aneusomies and aneuploidies of chromosomes. Aneusomies and aneuploidies of all chromosome arms are reported. The *red square* indicates a non-mosaic gain (log2 ratio > 0.6), the *pink square* a mosaic gain, and the *green square* a mosaic loss. Non-mosaic losses were not found. The *yellow square* indicates a gain or a loss which includes 50–80 % of the chromosome arm; its absence indicates aberration larger than 80 % of the chromosome arm. *Numbers reported in square* expresses the percentage of mosaicism

The most frequent aneuploidies in tumoral samples were as follows: loss of 18q (10/15 cases, all mosaic); loss of whole chromosome 18 (8 cases, all mosaic); gain of 20q (8 cases, 5 non-mosaic, and 3 mosaic); gain of whole chromosome 20 (7 cases, 3 non-mosaic, and 4 mosaic); gain of 8q (7 cases, 3 non-mosaic, and 4 mosaic); gain of 13q (7 cases, 4 non-mosaic, and 3 mosaic); loss of 17p (7 cases, all mosaic); gain of whole chromosome 7 (6 cases, 1 non-mosaic, and 5 mosaic) (Fig. 5).

Also, combinations of different aneuploidies were found, such as both loss of chr18 and gain of chr20 (7 cases) and both gains of chr13 and chr20 (4 cases). One case showed chromothripsis on chromosome 13 and 15. No aneuploidies were found in the normal samples.

#### Loss of heterozygosity

The slides used in our analysis combine array-CGH based CNA detection with fully research-validated SNP content, allowing confident and cost-effective CNA and loss of heterozygosity (LOH) identification using a single array (http://www.ogt.co.uk).

**Fig. 6:**
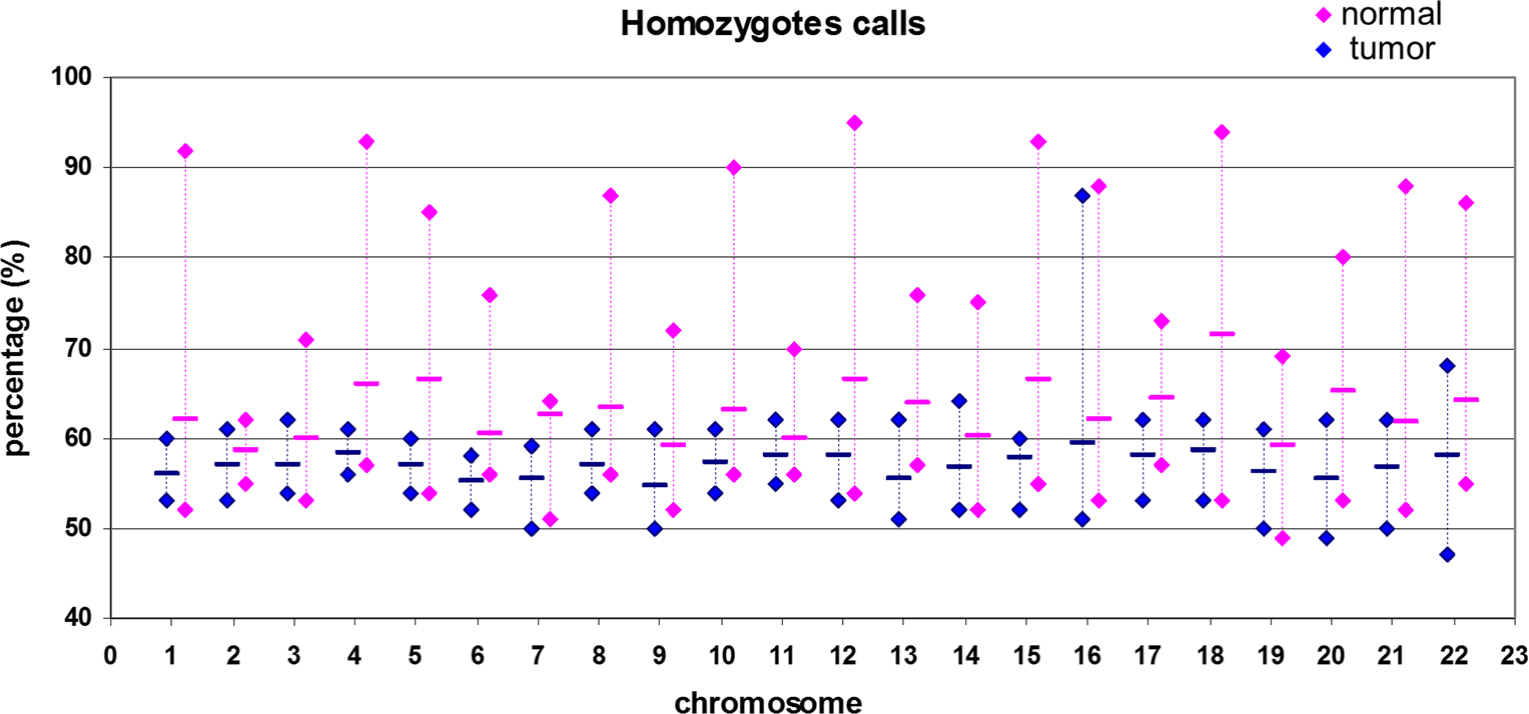
Homozygote calls. Percentage of homozygote calls for each chromosome in normal (*blue*) and tumoral (*pink*) samples

Analysis of the percentage of homozygote calls for each chromosome in different samples highlighted a very narrow distribution of percentages in the normal samples, ranging from 47 to 68 % for all chromosomes, except for chromosome 16, the only one whose distribution was comparable in both normal and tumoral samples. At variance, a larger distribution for all chromosomes, except for chromosome 2, was found in tumoral samples. The highest percentages were reached by chromosomes 12, 18, 4, and 15, whereas the highest average was that of chromosome 18, followed by chromosomes 5, 12, and 15 (Fig. 6).

Moreover, tumors showed a higher number of LOH regions: in fact, only 4/15 normal samples showed LOH regions, compared to 11/15 tumoral samples. We then selected shared LOH region in normal and tumoral samples for further analysis (Tables 4 and 5) and looked for copy neutral vs copy gain LOH, being the first defined as an LOH accompanied by disomy (cnLOH) and the latter an LOH accompanied by a gain (copy gain LOH).

**Table 4:** Shared normal LOH

Case	LOH	Copy number
1 5	Xp21.3(25,201,471–27,670,674) Xp21.3(25,617,005–27,839,601)	Disomy Disomy
5 12	Xq26.2-q26.3(131,154,109–135,439,567) Xq26.2(131,850,257–132,564,169)	Disomy Disomy

**Table 5:** Shared tumoral LOH

Case	LOH	Copy number
5 7 7 11 14 14 14	5q31.1-q31.3(133,199,222–141,279,379) 5q13.2-q21.2(70,853,813–102,979,449) 5q21.2-q35.3(103,478,488–179,995,873) 5q35.1-q35.3(171,218,108–179,995,873) 5q13.2-q14.3(71,697,947–92,105,488) 5q15-q31.1(94,050,457–135,052,084) 5q31.2-q35.1(136,772,653–170,057,632)	Disomy Disomy Disomy Disomy Disomy Disomy Disomy
8 11 15	13q32.3-q33.3(99,618,092–107,338,830) 13q33.1(101,706,686–103,566,689) 13q32.1-q33.2(97,931,679–105,224,699)	Gain Gain Gain
1 5 7	Xp21.3(25,201,471–27,670,674) Xp21.3(25,617,005–27,839,601) Xp22.2-p21.1(14,609,911–36,404,916)	Gain Disomy Gain
1 1 7 9 11	Xq21.1-q21.2(81,575,613–85,350,400) Xq21.32-q22.2(93,471,828–103,105,091) Xq21.31-q22.3(90,309,798–104,454,079) Xq21.1-q22.3(81,575,613–104,454,079) Xq21.1-q22.1(81,575,613–101,407,750)	Disomy Disomy Gain Gain Gain and disomy
1 1 5 6 7 9 11 12	Xq23-q25(114,667,580–123,679,482) Xq26.2-q28(132,406,770–153,390,510) Xq26.2-q26.3(131,154,109–136,075,759) Xq26.3-q28(133,706,195–153,390,510) Xq23-q28(111,782,809–153,390,510) Xq23-q28(111,782,809–153,390,510) Xq23-q28(111,722,801–153,390,510) Xq26.2(130,969,464–133,339,719)	Disomy Disomy Disomy Disomy and gain Gain Gain Gain Gain

In normal tissues (samples 1 and 5), we found a copy-neutral LOH on region Xp21.3 which contains the *MAGEB18*, *MAGEB6*, and *MAGEB5* genes, already reported in gain in two other normal samples. In tumoral samples, the copy-neutral LOH of 5q was shared by four cases, whereas shared LOHs of chromosome 13q, coinciding with gains of these regions, indicated that there are multiple copies of a single chromosome 13. Finally, many samples shared LOH on Xq regions, several as copy-neutral LOH and others as gains.

LOH corresponding to the copy number loss are not reported because the concordance confirms deletion of interested region.

#### Age and origin

Next, we analyzed a possible correlation between the age of patients and CNAs. In normal samples, a decrease of CNAs in patients less than 65 years and a small increase in those over 65 years was found, similar to those reported in literature for tumoral samples (Fig. S3). At variance in tumoral samples, a small increase under 65 years and a strong decrease over 65 years was observed.

Moreover, we analyzed the origin of the samples and noticed that normal and tumoral samples coming from one single hospital showed a higher (but not statistically significant) number of CNAs when compared to those coming from the other centers (Fig. S4).

## Discussion

Colorectal cancer is a highly heterogeneous disease and for this reason, several genomic studies have been performed, always analyzing in detail tumoral tissues using paired normal tissues as reference for the tumoral counterpart. Only one paper so far reported small gains at 19q and 20q in pooled normal tissues [9]. However, tumorigenic genetic lesions may be already present also when the colonic tissue appear histologically normal, as suggested by the fact that in 10–20 % of CRC patients undergoing surgery with curative intent, a local recurrence successively occurs [10]. In strong support to this suggestion, our data revealed for the first time an unexpected frequency of genetic alterations in histologically normal colonic tissue.

Accordingly, a very recent paper reported that transcriptional profiles of paired normal samples could be of great interest to better understand tumorigenesis mechanisms and progression and identify potential therapeutic targets [14].

Our approach of performing CGH + SNP Microarray on 15 paired histologically normal and tumoral samples allowed us to find a high number of CNAs (average: 160) also in normal colonic tissues. Our experiments showed excellent DLRS values, so also mosaic CNAs were included in the study with a good confidence. The possibility to detect mosaic CNAs allows obtaining a representation of all sample subpopulations, not only the most represented. For this reason, we found a higher number of CNAs compared to some previous studies.

Mosaicism is defined as the occurrence of more than one genetically diverse cell population in an organism arising from a single fertilized egg [15]. Somatic mosaicism is a typical condition of cancer, which is a mixture of different subpopulations.

In normal tissues many of affected genes involved or claimed to be related to the tumorigenic process. For example, the *RUNX1* gene is considered a tumor suppressor gene in myeloid neoplasm and was recently implicated in gastrointestinal tumorigenesis, as a novel tumor-suppressor gene, which maintains the balance between the intestinal stem/progenitor cell population and epithelial differentiation [16]. Moreover, genetic variations in *RUNX1* were associated with colorectal cancer risk [17].

Surprisingly, other tumor-suppressor genes, such as *PTCH1*, *DAB2IP*, and *WT1* were included in the gained regions. Lower levels of *PTCH1* mRNA were associated to high-risk for metastasis [18], and aberrant methylation of the *PTCH1* promoter might be an early event of colon carcinogenesis [19], thus leaving the meaning of this gain worthy to be further investigated. Notably, *WT1* may act both as a tumor suppressor or oncogene in different context [20]. In colorectal adenocarcinoma, it has been reported to be overexpressed [21] and its expression being a marker of poor prognosis [22].

Traditionally, histologically normal tissue has been considered to be appropriate control for copy number studies as it was not thought to have copy number aberrations: unexpectedly, instead, our data showed several oncogenes with copy number gain. Overexpression of *TRIB2*, resulting from gene amplification, has been described in lung cancer with a potential role in tumorigenesis [23]. *RET* promoter CpG island methylation is associated with a poor prognosis in stage II colorectal cancer [24], but *RET* fusion kinases has also been identified [25]. Moreover, *RET* CNAs has been reported in colorectal cancer [26]. Conversely, *MPL*, *TRIB2*, and *ARAF* copy number gains have not previously been identified in colon samples.

Finally, a mosaic gain on 8q24.21 containing *MYC* oncogene was found in two normal tissues, whose matched tumors have an undifferentiated histotype. Since this gain has not been found in other normal samples, we put forward the hypothesis that it may be considered as an early alteration for this tumor histotype.

As for normal tissues, also in tumoral samples, we found CNAs containing *RET* gene, both in gain and in loss, as previously reported [26]. Also, a shared CNA containing *APP* gene was both in gain and in loss in different samples. Recently, increased expression of *APP* in several types of cancer has been reported and it has been implicated in proliferation and motility of advanced breast cancer [27]. To our knowledge, this is the first time that *APP* CNAs is shown in cancer.

We speculated that CNAs present only in tumors and disomic in normal samples might have a possible role in tumor progression. For example, in tumoral tissues, we found two gained regions shared among at least three samples and containing *RBFOX1* and *PCDH11Y* genes. *RBFOX1* belongs to a family of evolutionarily conserved RNA-binding proteins and regulates tissue-specific alternative splicing: deletion of *RBFOX1* has been already reported in colon tumors [28] whereas copy number gain has been recently shown [29]. *PCDH11Y* instead plays a role during development of the central nervous system, but it has also been reported to be involved in prostate cancer progression [30]. Ours is the first finding that *PCDH11Y* can be present in an altered copy number in CRC.

As already noted, the use in array-CGH analysis of normal tissue as a reference for the corresponding tumor allows uncovering only alterations not equally represented in both tissue, leaving equal ones undetected and implying that CNAs present in both tissues are more amplified in the tumor; otherwise, they would have been scored as disomic.

The identified gained regions contain *TRIB2*, *MFI2*, *RET*, *WNT1*, *DDN*, *PRKAG1*, *KMT2D*, *RUNX1*, *BRWD3*, *GRIA3*, and *XIAP*. All these genes are known to be involved, at least to a certain degree, in different types of cancer: based on our findings, we suggest that they might have a critical role also in colorectal cancer onset and progression. Moreover, some of these genes are target of current clinical trials for other types of cancer, such as *RET* for non-small cell lung cancer, *RUNX1* for leukemia, and *XIAP* for pancreatic and breast cancers (http://clinicaltrials.gov, https://www.clinicaltrialsregister.eu), so they could be interesting targets also for colorectal cancer therapy.

Tumor copy number changes were also compared with data of the Cancer Genomic Atlas (TCGA) database, where all tumors were paired with normal tissue specimen which could be blood/blood components, adjacent normal tissue, or previously extracted germline DNA from blood [31]. In the TCGA database, the most frequent aneuploidies are gains of 1q, 7, 8 12q, 13q, and 20 and losses of 18 (66 % of cases), 17 (56 %), followed by 1p, 4q, 5q, 8p, 14, 15, 20p, and 22q. Their data are in agreement with our results as evident from Fig. 5 and the “Results” section; especially but not only for loss of 18 (66 % of our cases) and for loss of 17p (46 %).

Loss of heterozygosity (LOH) occurs in a variety of tumor types and is considered important for tumor progression. After the loss of one allele, the remaining allele can be affected by somatic mutation or harbor a disease-prone polymorphic variant [32]. It may happen that the lost region is replaced by an exact copy of the remaining chromosome, resulting in the retention of two copies of genetic information, but the loss of polymorphic differences [32]; in this case, we speak about copy-neutral LOH (also called uniparental disomy).

Analysis of homozygote calls for each chromosome revealed a strong difference in LOH regions between normal and tumoral samples.

Normal samples showed a lower number of LOH compared to tumoral samples, being only two copy-neutral LOHs shared between two different samples and located in Xp21.3 and Xq26.2. The Xp21.3 region contains *MAGE* family genes and interestingly, it is observed in copy number gain in two other normal samples. It is possible to assume that this region may be subject to genomic alterations in normal samples.

Copy-neutral LOH was found on chromosome 5q for four tumoral cases. LOH of 5q was reported in ovarian cancer, frequently accompanied by *TP53* mutation [33]. It was also observed in adenoma samples, suggesting that it may play an important role in tumor initiation [34]. In 5q region maps *APC* gene. *APC* LOH was found in four samples accompanied by copy number loss in two cases and by the presence of two copies of the same regions in the other two samples; this identifies a copy neutral LOH. According to these data, a recent paper reported that most of sporadic colorectal carcinomas with LOH for *APC* return to copy number neutrality with the duplication of the remaining allele [35]. The copy gain LOH of 13q33.1 is shared by three tumoral cases and indicates multiple copies of the same allele.

CNAs were also compared with age of patients and samples’ origin. It was reported that humans are commonly affected by somatic mosaicism for stochastic CNAs. The rate of CNA occurrence may differ between cell types or may be related to the age of individual cells [36]. We found that the number of CNAs in normal tissues did not increase linearly with the age as reported in literature for tumoral samples [37], whereas our tumoral tissues showed an opposite trend. These results could be explained by the fact that our tumoral tissues were analyzed against the corresponding normal sample and not against a reference. Analyzing the origin of samples we found an increased, even though non-significant, number of CNAs especially in normal samples coming from a single hospital, leading us to hypothesize that this increase may be due to a possible environmental effect.

In conclusion, our approach to perform CGH + SNP Microarray on paired normal and tumoral samples, allowed us to uncover for the first time an unexpected frequency of genetic alteration in normal colonic tissue from colon cancer patients, suggesting that (1) tumorigenic genetic lesions are already present also when the colonic tissue appear histologically normal; (2) the use in array-CGH studies of normal samples as reference for the paired tumors can lead to misrepresented genomic data, due the loss of identical CNAs between the two samples; and (3) even available array-CGH data may be incomplete or limited, especially if used for the research of target molecules for personalized therapy and for the possible correlation with clinical outcome.

## Electronic supplementary material

Table S1 Shared copy number alterations in normal samples. (XLSX 16 kb)

Table S2 Statistically over-represented GO terms in gained genes. (DOC 64 kb)

Fig S1 Statistically over-represented GO terms in gained genes of normal samples. The most represented classes of GO terms are reported, divided into three categories: biological process, molecular function and cellular component. (GIF 34 kb)

High resolution image (TIFF 247 kb)

Fig S2 Examples of graphical representation of log2 ratio averages and standard deviations of chromosome probes (case 1). This figure was automatically produced by CytoSure Interpret Software and showed log2 ratio averages and standard deviations for all chromosomes in patient 1 normal and tumoral samples. (GIF 231 kb)

High resolution image (TIFF 2302 kb)

Fig S3 Distribution of CNAs according to patients’ age. Blu dots indicate the number of CNAs in normal samples, pink dots in tumoral samples and green dots in tumoral samples reported in the literature. Lines indicate the trend line. (GIF 27 kb)

High resolution image (TIFF 363 kb)

Fig S4 Distribution of CNAs according to different hospitals. Box plots representation of copy number alterations in different hospitals. The first and third quartiles are at the ends of the box, the median is indicated with an inside line of the box, and the maximum and minimum value are indicated with dots. (GIF 17 kb)

High resolution image (TIFF 287 kb)

## Abbreviations

CGH: comparative genomic hybridization
CIMP: CpG island methylator phenotype
CIN: chromosomal instability
CNA: copy number alteration
CRC: colorectal cancer
LOH: loss of heterozygosity
MSI: microsatellite instability

## References

[1] Worthley DL, Leggett BA (2010). Colorectal cancer: molecular features and clinical opportunities. Clin Biochem Rev.

[2] Ried T, Knutzen R, Steinbeck R, Blegen H, Schröck E, Heselmeyer K, du Manoir S, Auer G (1996). Comparative genomic hybridization reveals a specific pattern of chromosomal gains and losses during the genesis of colorectal tumors. Genes Chromosom Cancer.

[3] Douglas EJ, Fiegler H, Rowan A, Halford S, Bicknell DC, Bodmer W, Tomlinson IP, Carter NP (2004). Array comparative genomic hybridization analysis of colorectal cancer cell lines and primary carcinomas. Cancer Res.

[4] Diep CB, Kleivi K, Ribeiro FR, Teixeira MR, Lindgjaerde OC, Lothe RA (2006). The order of genetic events associated with colorectal cancer progression inferred from meta-analysis of copy number changes. Genes Chromosom Cancer.

[5] Lagerstedt KK, Kristiansson E, Lönnroth C, Andersson M, Iresjö BM, Gustafsson A, Hansson E, Kressner U, Nordgren S, Enlund F, Lundholm K (2010). Genes with relevance for early to late progression of colon carcinoma based on combined genomic and transcriptomic information from the same patients. Cancer Informat.

[6] Orsetti B, Selves J, Bascoul-Mollevi C, Lasorsa L, Gordien K, Bibeau F, Massemin B, Paraf F, Soubeyran I, Hostein I, Dapremont V, Guimbaud R, Cazaux C, Longy M, Theillet C (2014). Impact of chromosomal instability on colorectal cancer progression and outcome. BMC Cancer.

[7] Nakao M, Kawauchi S, Furuya T, Uchiyama T, Adachi J, Okada T, Ikemoto K, Oga A, Sasaki K (2009). Identification of DNA copy number aberrations associated with metastases of colorectal cancer using array cgh profiles. Cancer Genet Cytogenet.

[8] Jasmine F, Rahaman R, Dodsworth C, Roy S, Paul R, Raza M, Paul-Brutus R, Kamal M, Ahsan H, Kibriya MG (2012). A genome-wide study of cytogenetic changes in colorectal cancer using snp microarrays: opportunities for future personalized treatment. PLoS One.

[9] Lagerstedt KK, Staaf J, Jönsson G, Hansson E, Lönnroth C, Kressner U, Lindström L, Nordgren S, Borg A, Lundholm K (2007). Tumor genome wide dna alterations assessed by array cgh in patients with poor and excellent survival following operation for colorectal cancer. Cancer Informat.

[10] Young PE, Womeldorph CM, Johnson EK, Maykel JA, Brucher B, Stojadinovic A, Avital I, Nissan A, Steele SR (2014). Early detection of colorectal cancer recurrence in patients undergoing surgery with curative intent: current status and challenges. J Cancer.

[11] Cheung SW, Shaw CA, Scott DA, Patel A, Sahoo T, Bacino CA, Pursley A, Li J, Erickson R, Gropman AL, Miller DT, Seashore MR, Summers AM, Stankiewicz P, Chinault AC, Lupski JR, Beaudet AL, Sutton VR (2007). Microarray-based CGH detects chromosomal mosaicism not revealed by conventional cytogenetics. Am J Med Genet A.

[12] Beissbarth T, Speed TP (2004). Gostat: find statistically overrepresented gene ontologies within a group of genes. Bioinformatics.

[13] Wang H, Liang L, Fang JY, Xu J Somatic gene copy number alterations in colorectal cancer: new quest for cancer drivers and biomarkers. Oncogene 2015 10.1038/onc.2015.304 26257062

[14] Huang X, Stern DF, Zhao H (2016). Transcriptional profiles from paired normal samples offer complementary information on cancer patient survival - evidence from tcga pan-cancer data. Sci Rep.

[15] Youssoufian H, Pyeritz RE (2002). Mechanisms and consequences of somatic mosaicism in humans. Nat Rev Genet.

[16] Fijneman RJ, Anderson RA, Richards E, Liu J, Tijssen M, Meijer GA, Anderson J, Rod A, O’Sullivan MG, Scott PM, Cormier RT (2012). Runx1 is a tumor suppressor gene in the mouse gastrointestinal tract. Cancer Sci.

[17] Slattery ML, Lundgreen A, Herrick JS, Caan BJ, Potter JD, Wolff RK (2011). Associations between genetic variation in runx1, runx2, runx3, mapk1 and eif4e and risk of colon and rectal cancer: additional support for a tgf-β-signaling pathway. Carcinogenesis.

[18] You S, Zhou J, Chen S, Zhou P, Lv J, Han X, Sun Y (2010). Ptch1, a receptor of hedgehog signaling pathway, is correlated with metastatic potential of colorectal cancer. Ups J Med Sci.

[19] Peng L, Hu J, Li S, Wang Z, Xia B, Jiang B, Li B, Zhang Y, Wang J, Wang X (2013). Aberrant methylation of the ptch1 gene promoter region in aberrant crypt foci. Int J Cancer.

[20] Yang L, Han Y, Suarez Saiz F, Saurez Saiz F, Minden MD (2007). A tumor suppressor and oncogene: the wt1 story. Leukemia.

[21] Oji Y, Yamamoto H, Nomura M, Nakano Y, Ikeba A, Nakatsuka S, Abeno S, Kiyotoh E, Jomgeow T, Sekimoto M, Nezu R, Yoshikawa Y, Inoue Y, Hosen N, Kawakami M, Tsuboi A, Oka Y, Ogawa H, Souda S, Aozasa K, Monden M, Sugiyama H (2003). Overexpression of the wilms’ tumor gene wt1 in colorectal adenocarcinoma. Cancer Sci.

[22] Bejrananda T, Phukaoloun M, Boonpipattanapong T, Wanitsuwan W, Kanngern S, Sangthong R, Sangkhathat S (2010). Wt1 expression as an independent marker of poor prognosis in colorectal cancers. Cancer Biomark.

[23] Grandinetti KB, Stevens TA, Ha S, Salamone RJ, Walker JR, Zhang J, Agarwalla S, Tenen DG, Peters EC, Reddy VA (2011). Overexpression of trib2 in human lung cancers contributes to tumorigenesis through downregulation of c/ebpα. Oncogene.

[24] Draht MX, Smits KM, Tournier B, Jooste V, Chapusot C, Carvalho B, Cleven AH, Derks S, Wouters KA, Belt EJ, Stockmann HB, Bril H, Weijenberg MP, van den Brandt PA, de Bruïne AP, Herman JG, Meijer GA, Piard F, Melotte V, van Engeland M (2014). Promoter cpg island methylation of ret predicts poor prognosis in stage ii colorectal cancer patients. Mol Oncol.

[25] Le Rolle AF, Klempner SJ, Garrett CR, Seery T, Sanford EM, Balasubramanian S, Ross JS, Stephens PJ, Miller VA, Ali SM, Chiu VK (2015). Identification and characterization of ret fusions in advanced colorectal cancer. Oncotarget.

[26] Brim H, Abu-Asab MS, Nouraie M, Salazar J, Deleo J, Razjouyan H, Mokarram P, Schaffer AA, Naghibhossaini F, Ashktorab H (2014). An integrative cgh, msi and candidate genes methylation analysis of colorectal tumors. PLoS One.

[27] Lim S, Yoo BK, Kim HS, Gilmore HL, Lee Y, Lee HP, Kim SJ, Letterio J, Lee HG (2014). Amyloid-β precursor protein promotes cell proliferation and motility of advanced breast cancer. BMC Cancer.

[28] Sengupta N, Yau C, Sakthianandeswaren A, Mouradov D, Gibbs P, Suraweera N, Cazier JB, Polanco-Echeverry G, Ghosh A, Thaha M, Ahmed S, Feakins R, Propper D, Dorudi S, Sieber O, Silver A, Lai C (2013). Analysis of colorectal cancers in British Bangladeshi identifies early onset, frequent mucinous histotype and a high prevalence of rbfox1 deletion. Mol Cancer.

[29] Mampaey E, Fieuw A, Van Laethem T, Ferdinande L, Claes K, Ceelen W, Van Nieuwenhove Y, Pattyn P, De Man M, De Ruyck K, Van Roy N, Geboes K, Laurent S (2015). Focus on 16p13.3 locus in colon cancer. PLoS One.

[30] Terry S, Queires L, Gil-Diez-de-Medina S, Chen MW, de la Taille A, Allory Y, Tran PL, Abbou CC, Buttyan R, Vacherot F (2006). Protocadherin-pc promotes androgen-independent prostate cancer cell growth. Prostate.

[31] Network CGA (2012). Comprehensive molecular characterization of human colon and rectal cancer. Nature.

[32] Makishima H, Maciejewski JP (2011). Pathogenesis and consequences of uniparental disomy in cancer. Clin Cancer Res.

[33] Tavassoli M, Steingrimsdottir H, Pierce E, Jiang X, Alagoz M, Farzaneh F, Campbell IG (1996). Loss of heterozygosity on chromosome 5q in ovarian cancer is frequently accompanied by tp53 mutation and identifies a tumour suppressor gene locus at 5q13.1-21.. Br J Cancer.

[34] Zarzour P, Boelen L, Luciani F, Beck D, Sakthianandeswaren A, Mouradov D, Sieber OM, Hawkins NJ, Hesson LB, Ward RL, Wong JW (2015). Single nucleotide polymorphism array profiling identifies distinct chromosomal aberration patterns across colorectal adenomas and carcinomas. Genes Chromosom Cancer.

[35] Zauber P, Marotta S, Sabbath-Solitare M (2016). Copy number of the adenomatous polyposis coli gene is not always neutral in sporadic colorectal cancers with loss of heterozygosity for the gene. BMC Cancer.

[36] Piotrowski A, Bruder CE, Andersson R, Diaz de Ståhl T, Menzel U, Sandgren J, Poplawski A, von Tell D, Crasto C, Bogdan A, Bartoszewski R, Bebok Z, Krzyzanowski M, Jankowski Z, Partridge EC, Komorowski J, Dumanski JP (2008). Somatic mosaicism for copy number variation in differentiated human tissues. Hum Mutat.

[37] Brim H, Lee E, Abu-Asab MS, Chaouchi M, Razjouyan H, Namin H, Goel A, Schäffer AA, Ashktorab H (2012). Genomic aberrations in an African American colorectal cancer cohort reveals a msi-specific profile and chromosome x amplification in male patients. PLoS One.

