# Supplementary material for: Unexpected frequency of genomic alterations in histologically normal colonic tissue from colon cancer patients
Source: Tumour Biol. 2016 Aug 2;37(10):13831–42. doi: 10.1007/s13277-016-5181-0 (PMC5097093; doi:10.1007/s13277-016-5181-0)
Supplement: Supplementary file 2 — Statistically over-represented GO terms in gained genes. (DOC 64 kb) [file 13277_2016_5181_MOESM2_ESM.doc]

**Table S2: Statistically over-represented GO terms in gained genes.**

| **BIOLOGICAL PROCESS** | **CELLULAR PROCESS** (105) | cell cycle (9) |
| --- | --- | --- |
|  |  | cellular metabolic process (29) |
|  |  | cell proliferation (2) |
|  |  | cell communication (17) |
|  |  | cellular component organiz. and biogenesis (9) |
|  |  | cell development (17) |
|  |  | cell activation (13) |
|  |  | cellular developmental process (4) |
|  |  | cell motility (2) |
|  |  | cell recognition (3) |
|  | **DEVELOPMENTAL PROCESS** (34) | anatomical structure development (26) |
|  |  | multicellular organismal development (6) |
|  |  | death (1) |
|  |  | developmental process (1) |
|  | **BIOLOGICAL REGULATION**  (134) | regulation of biological quality (7) |
|  |  | regulation of a molecular function (17) |
|  |  | regulation of biological process (109) |
|  |  | biological regulation (1) |
|  | **MULTICELLULAR ORGANISMAL PROCESS** (10) | system process (3) |
|  |  | multicellular organism reproduction (4) |
|  |  | cytokine production (2) |
|  |  | multicellular organismal process (1) |
|  | **RESPONSE TO STIMULUS** (12) | response to external stimulus (3) |
|  |  | response to stress (1) |
|  |  | response to biotic stimulus (1) |
|  |  | response to chemical stimulus (3) |
|  |  | defense response (3) |
|  |  | behavior (1) |
|  | **METABOLIC PROCESS** (5) | primary metabolic process (1) |
|  |  | nitrogen compound metabolic process (1) |
|  |  | macromolecule metabolic process (3) |
|  | **ESTABLISHMENT OF LOCALIZATION** (5) | transport (3) |
|  |  | secretion (1) |
|  |  | localization (1) |
|  | **REPRODUCTION** (5) | sexual reproduction (2) |
|  |  | reproductive process (3) |
|  | **BIOLOGICAL ADHESION**  (3) |  |
| **MOLECULAR FUNCTION** | **BINDING** (23) | nucleic acid binding (3) |
|  |  | protein binding (13) |
|  |  | nucleotide binding (4) |
|  |  | nucleobase binding (3) |
|  | **CATALYTIC ACTIVITY** (24) | transferase activity (16)  hydrolase activity (6)  oxidoreductase activity (1)  isomerase activity (1) |
|  | **ENZYME REGULATOR ACTIVITY** (5) | kinase regulator activity (2) |
|  |  | enzyme inhibitor activity (3) |
|  | **TRANSPORTER ACTIVITY** (4) | substrate-specific transporter activity (2) |
|  |  | drug transporter activity (2) |
|  | **MOLECULAR TRANSDUCER ACTIVITY**  (4) |  |
| **CELLULAR COMPONENT** | **CELL PART** (40) | intracellular (23) |
|  |  | membrane (12) |
|  |  | cellular component (1) |
|  |  | cell fraction (2) |
|  |  | apical part of cell (2) |
|  | **EXTRACELLULAR MATRIX** (4) |  |
|  | **ORGANELLE** (4) |  |
|  | **MACROMOLECULAR COMPLEX** (3) |  |

Numbers of GO terms are in brackets.
