# Supplementary figures and images for: Unexpected frequency of genomic alterations in histologically normal colonic tissue from colon cancer patients
Source: Tumour Biol. 2016 Aug 2;37(10):13831–42. doi: 10.1007/s13277-016-5181-0 (PMC5097093; doi:10.1007/s13277-016-5181-0)

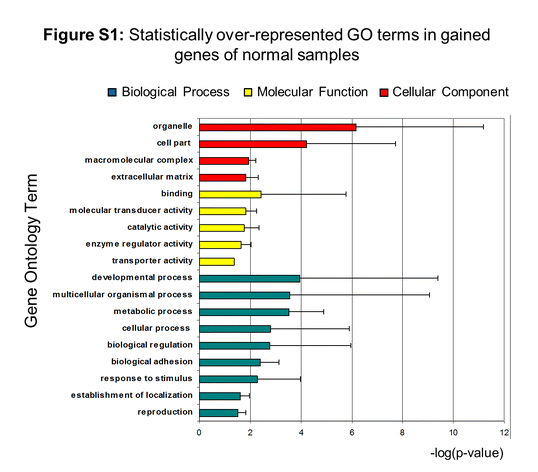

Supplement: Supplementary file 3 — Statistically over-represented GO terms in gained genes of normal samples. The most represented classes of GO terms are reported, divided into three categories: biological process, molecular function and cellular component. (GIF 34 kb) [file 13277_2016_5181_Fig7_ESM.gif]

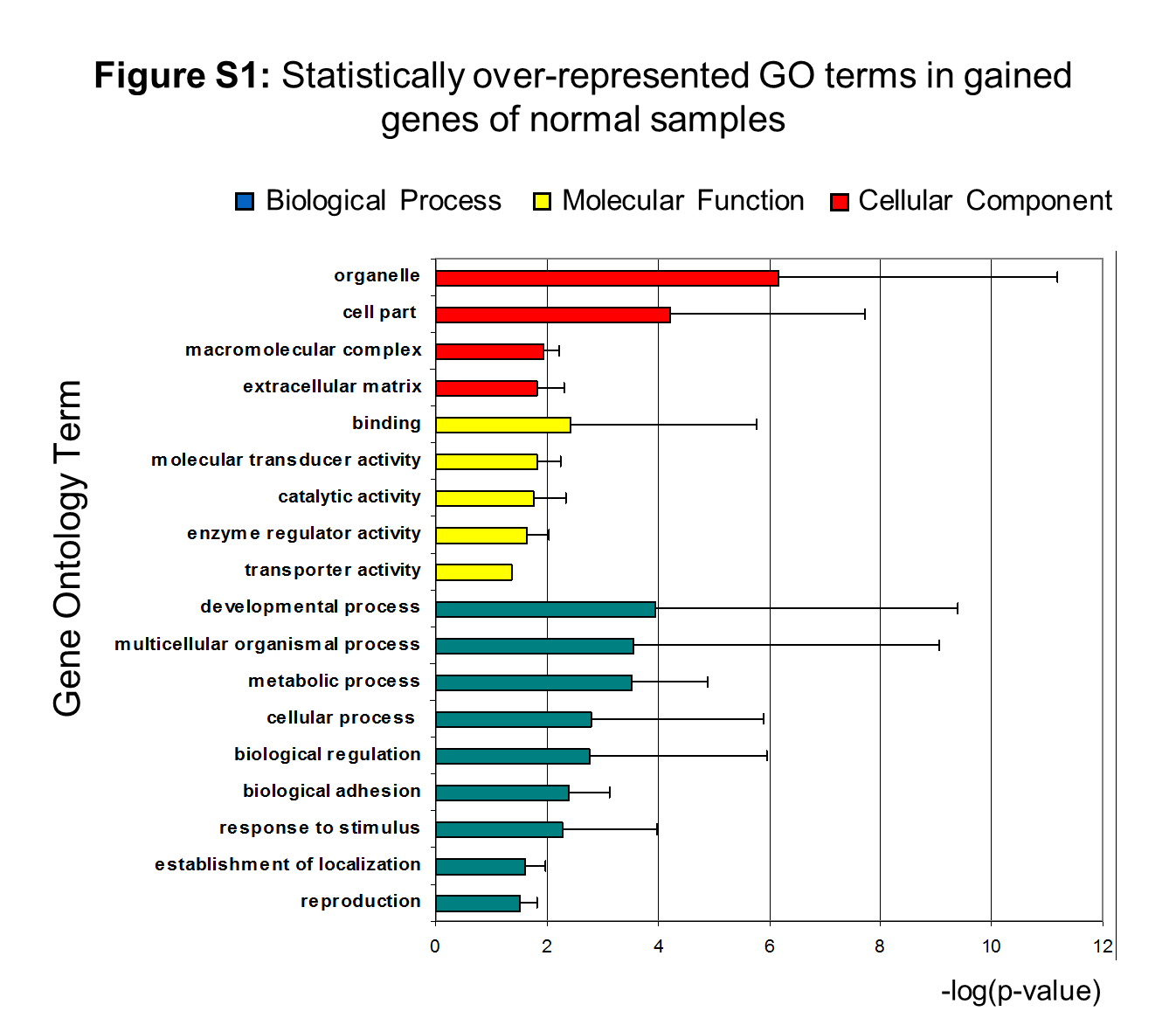

Supplement: Supplementary file 4 — High resolution image (TIFF 247 kb) [file 13277_2016_5181_MOESM3_ESM.tif]

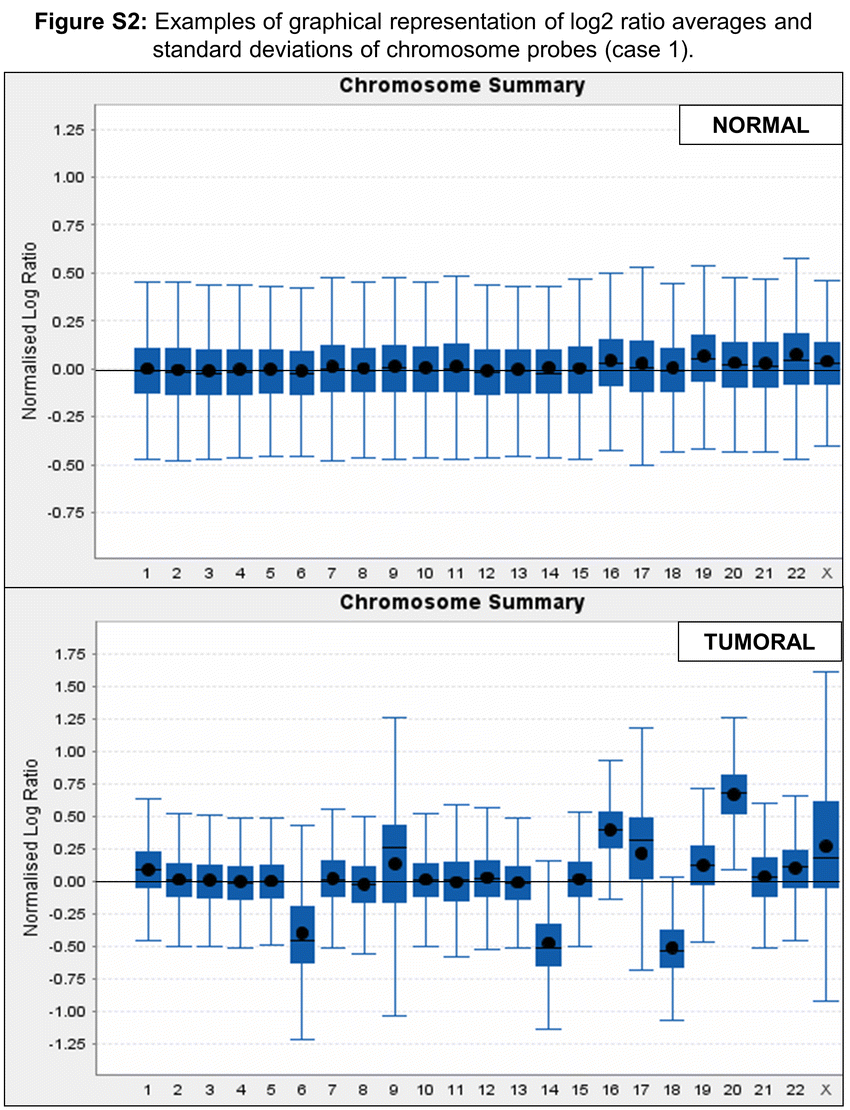

Supplement: Supplementary file 5 — Examples of graphical representation of log2 ratio averages and standard deviations of chromosome probes (case 1). This figure was automatically produced by CytoSure Interpret Software and showed log2 ratio averages and standard deviations for all chromosomes in patient 1 normal and tumoral samples. (GIF 231 kb) [file 13277_2016_5181_Fig8_ESM.gif]

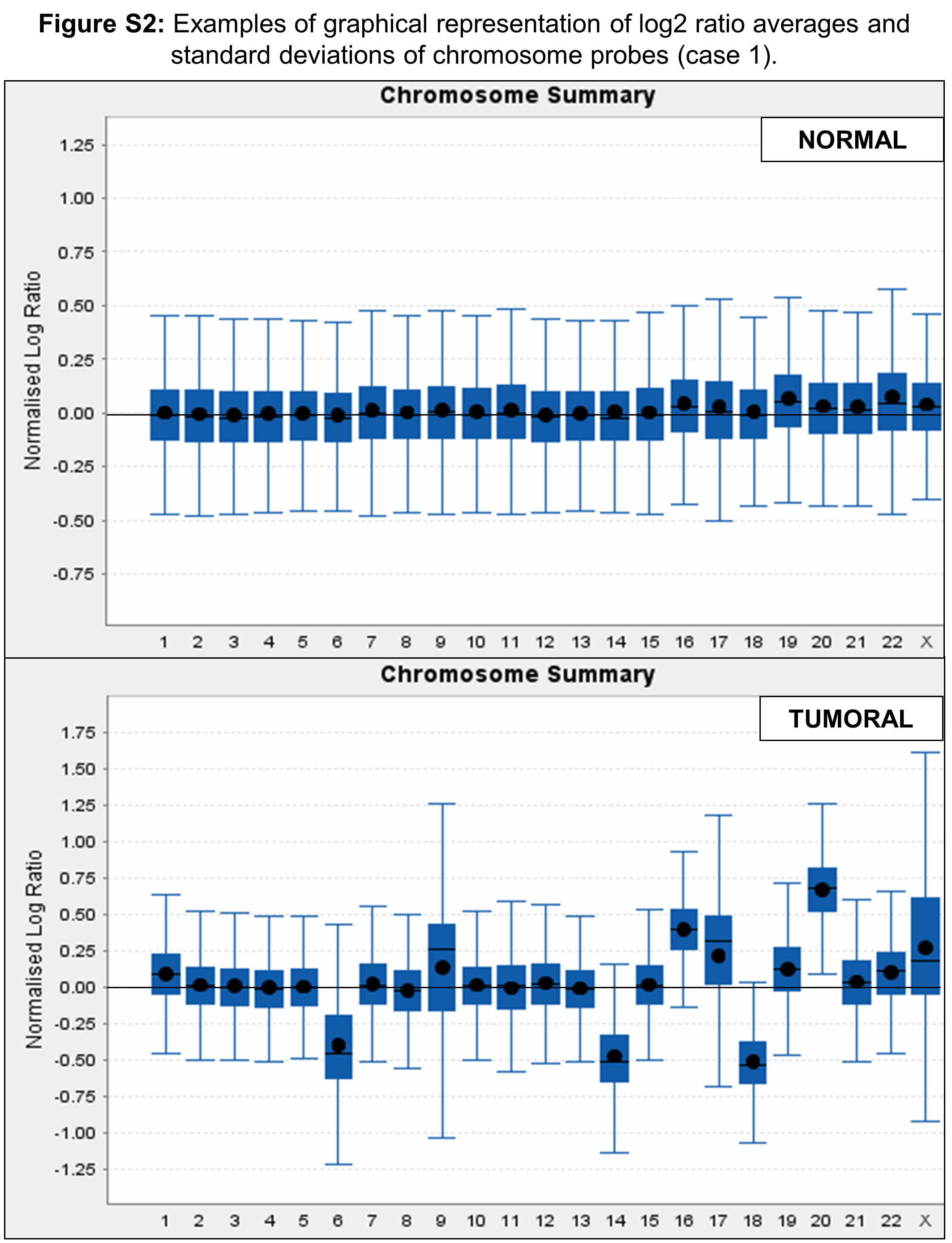

Supplement: Supplementary file 6 — High resolution image (TIFF 2302 kb) [file 13277_2016_5181_MOESM4_ESM.tif]

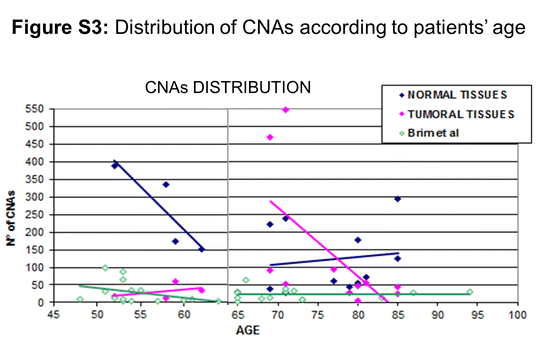

Supplement: Supplementary file 7 — Distribution of CNAs according to patients’ age. Blu dots indicate the number of CNAs in normal samples, pink dots in tumoral samples and green dots in tumoral samples reported in the literature. Lines indicate the trend line. (GIF 27 kb) [file 13277_2016_5181_Fig9_ESM.gif]

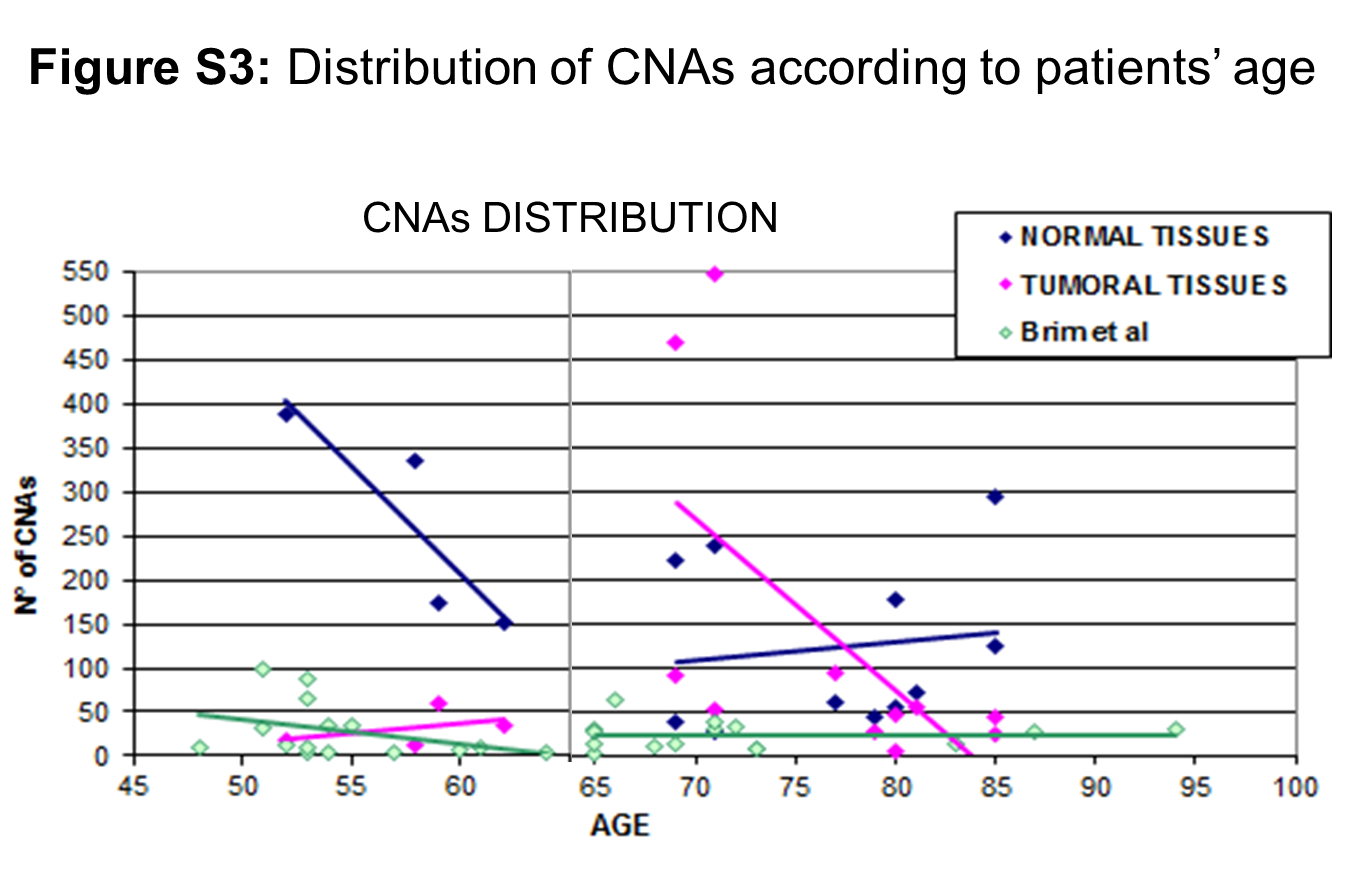

Supplement: Supplementary file 8 — High resolution image (TIFF 363 kb) [file 13277_2016_5181_MOESM5_ESM.tif]

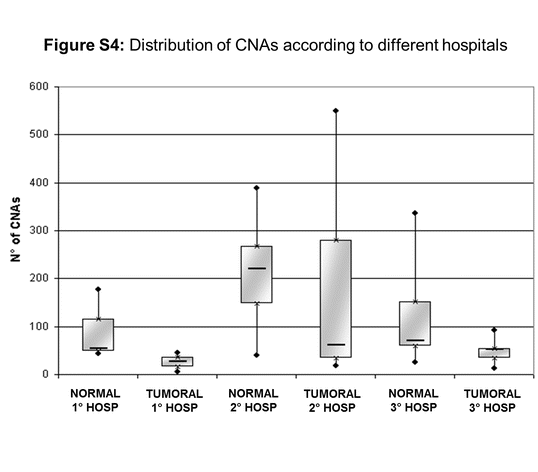

Supplement: Supplementary file 9 — Distribution of CNAs according to different hospitals. Box plots representation of copy number alterations in different hospitals. The first and third quartiles are at the ends of the box, the median is indicated with an inside line of the box, and the maximum and minimum value are indicated with dots. (GIF 17 kb) [file 13277_2016_5181_Fig10_ESM.gif]

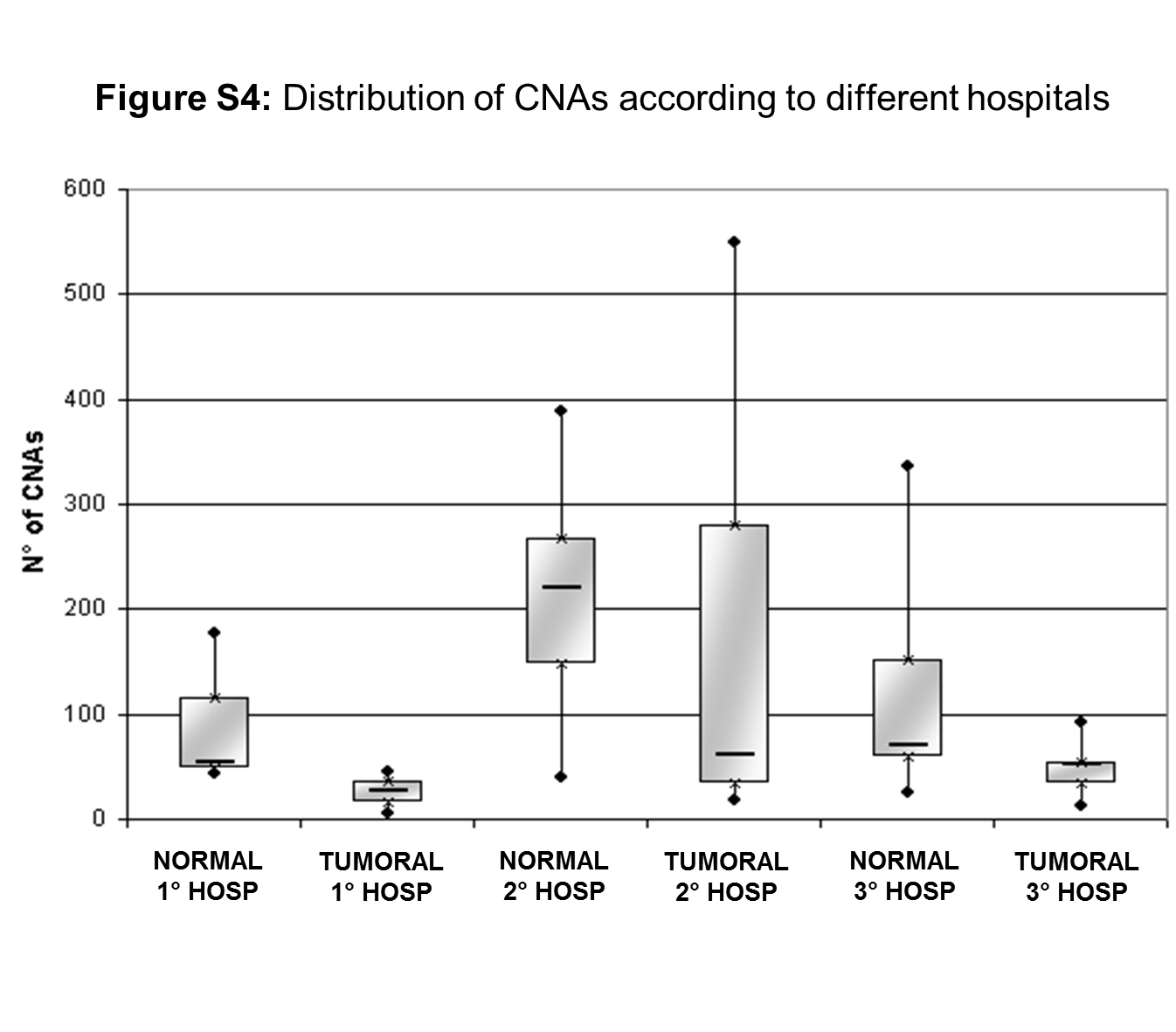

Supplement: Supplementary file 10 — High resolution image (TIFF 287 kb) [file 13277_2016_5181_MOESM6_ESM.tif]
